# Mechanomyography and Torque during FES-Evoked Muscle Contractions to Fatigue in Individuals with Spinal Cord Injury

**DOI:** 10.3390/s17071627

**Published:** 2017-07-14

**Authors:** Nor Zainah Mohamad, Nur Azah Hamzaid, Glen M. Davis, Ahmad Khairi Abdul Wahab, Nazirah Hasnan

**Affiliations:** 1 Department of Biomedical Engineering, Faculty of Engineering, University of Malaya, Kuala Lumpur 50603, Malaysia; jenazainah@siswa.um.edu.my (N.Z.M.); glen.davis@sydney.edu.au (G.M.D.); khairi@um.edu.my(A.K.A.W.); 2 Clinical Exercise and Rehabilitation Unit, Discipline of Exercise and Sports Sciences, Faculty of Health Sciences, University of Sydney, Lidcombe, NSW 2141, Australia; 3 Department of Rehabilitation Medicine, Faculty of Medicine, University of Malaya, Kuala Lumpur 50603, Malaysia; nazirah@ummc.edu.my

**Keywords:** MC sensor, spinal cord injury (SCI), muscle fatigue, functional electrical stimulation (FES)

## Abstract

A mechanomyography muscle contraction (MC) sensor, affixed to the skin surface, was used to quantify muscle tension during repetitive functional electrical stimulation (FES)-evoked isometric rectus femoris contractions to fatigue in individuals with spinal cord injury (SCI). Nine persons with motor complete SCI were seated on a commercial muscle dynamometer that quantified peak torque and average torque outputs, while measurements from the MC sensor were simultaneously recorded. MC-sensor-predicted measures of dynamometer torques, including the signal peak (SP) and signal average (SA), were highly associated with isometric knee extension peak torque (SP: r = 0.91, *p* < 0.0001), and average torque (SA: r = 0.89, *p* < 0.0001), respectively. Bland-Altman (BA) analyses with Lin’s concordance (*ρ*_C_) revealed good association between MC-sensor-predicted peak muscle torques (SP; *ρ*_C_ = 0.91) and average muscle torques (SA; *ρ*_C_ = 0.89) with the equivalent dynamometer measures, over a range of FES current amplitudes. The relationship of dynamometer torques and predicted MC torques during repetitive FES-evoked muscle contraction to fatigue were moderately associated (SP: r = 0.80, *p* < 0.0001; SA: r = 0.77; *p* < 0.0001), with BA associations between the two devices fair-moderate (SP; *ρ*_C_ = 0.70: SA; *ρ*_C_ = 0.30). These findings demonstrated that a skin-surface muscle mechanomyography sensor was an accurate proxy for electrically-evoked muscle contraction torques when directly measured during isometric dynamometry in individuals with SCI. The novel application of the MC sensor during FES-evoked muscle contractions suggested its possible application for real-world tasks (e.g., prolonged sit-to-stand, stepping,) where muscle forces during fatiguing activities cannot be directly measured.

## 1. Introduction

Muscle contractions, evoked by skin-surface functional electrical stimulation (FES) [1], result in rapid muscle fatigue, particularly during prolonged FES exercise performed by individuals with spinal cord injury (SCI), who lack voluntary muscle recruitment [2,3]. A limiting factor to any potential benefits of FES-evoked daily-living tasks has been rapid muscle fatigue [4], which leads to low adoption rates of FES-evoked activities by these individuals. Due to impaired proprioceptive responses that characterize SCI, it is essential to monitor muscular forces generated during FES-evoked movements [5,6]. Moreover, accurate real-time muscle force data is crucial for predicting the desired responses to FES-evoked activities, particularly when such activities are of prolonged duration, wherein rapid muscle fatigue is a limiting factor [7,8]. To date, muscle fatigue [9] has been quantified using sensors, such as surface electromyography (sEMG) [10,11], near-infrared spectroscopy (NIRS) [12] and mechanomyography (MMG) [13]. These body-worn sensors estimate muscle performance and muscle fatigue from metabolic responses, electrical signals, and mechanical properties of the contracting muscle, respectively [14]. Muscle responses derived from these sensors have revealed different characteristics during non-fatigued versus fatigued contractions [15]. A relatively novel muscle-contraction sensor (MC) was first introduced in 2011 [16,17] from the family of MMG-based sensors, and the MC-sensor is based on the principle of measuring skin-surface tension when a muscle is recruited. Attached to the skin surface over the muscle belly, the MC sensor tip exerts compression force onto the skin, in the intermediate layer over the muscle and the muscle itself [18]. The force measured at the tip of the sensor is deemed proportional to the muscle tension. Dordevic and colleagues [16] demonstrated that an MC sensor with ‘sensor tip’-applied pressure could accurately characterize muscle activity of the biceps brachii under isometric conditions compared to sEMG signals. A strong linear correlation between the evoked force and the MC signal (r = 0.97) suggested that the MC sensor could be an accurate proxy for muscle force in able-bodied individuals. While Đorđević et al. reported the use of the MC Sensor in able-bodied people, there has not yet been a study that addresses the use of FES, where involuntary contraction was induced with possible effect of electrical current interference to the signal. Therefore, the ability and sensitivity of the MC sensor for detecting electrically-induced muscle fatigue (i.e., a reduced force generating a capacity of muscle at an unchanged stimulation current) in persons with SCI remains an important and unanswered question. If the MC sensor can predict muscle torque accurately, it might be deployed in mission-critical daily functional tasks for neurological populations. On the other hand, the study by Zhang et al. [7] reported the use of EMG to predict torque production during FES induced muscle contraction. The application of EMG differs largely from the adoption of the MC sensor, in which the MC sensor uses a mechanically-based muscle-tension principle, while EMG measures electrical responses of the isometric evoked-muscle contraction [7].

The novelty of this study is in the detection of muscle force contraction evoked via electrical stimulation, thus bypassing the central nervous system using a mechanical-tension-based sensor that is applied directly to the skin surface over the muscle. This study sought to investigate MC sensor responses versus muscle torque measurements during prolonged, electrically-evoked, fatiguing muscle contractions in individuals with motor-complete SCI. Secondarily, whether the MC sensor could act as a body-worn proxy of directly-measured isometric knee extension torques derived from a commercial dynamometer was investigated.

## 2. Materials and Methods

### 2.1. Subjects

Nine healthy, spinal-cord injured (SCI) individuals (41.6 ± 14.5 year), with motor ‘complete’ lesions, based on Injury Classification of American Spinal Injuries Association Impairment Scale (AIS A and B between T7 and T11 neurological segments [19], volunteered to participate in this study. Their physical characteristics are shown in Table 1.

Participants were excluded if they presented with unstable angina pectoris, uncontrolled hypertension, or other medical conditions that would contraindicate medically safe participation in exercise. This study was approved by the University of Malaya Research Ethics Committee, UMREC (Approval No. 1003.14(1)), and all participants provided written informed consent after oral explanation of the procedures.

### 2.2. Instrumentation

A commercial neuromuscular stimulator (RehaStim2, Hasomed GmbH, Berlin, Germany) was used to generate electrical currents sufficient to recruit the participants’ rectus femoris muscles. To confirm that the rectus femoris was maximally recruited by electrical stimulation, when FES current was applied to the muscle, the rectus femoris muscle bulging was clearly visible and the rectus femoris evoked knee extension torque as monitored by the Biodex dynamometer.

**Figure 1:**
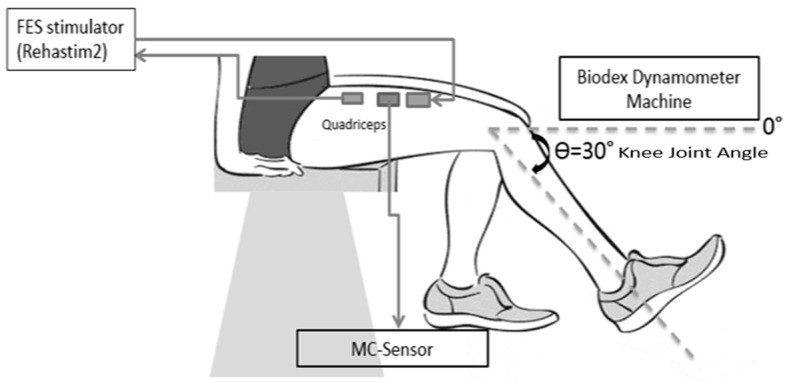
Instrumentation during FES-evoked muscle contractions using MC sensor with the angle of the knee joint set to 30° on Biodex Dynamometer.

**Figure 2:**
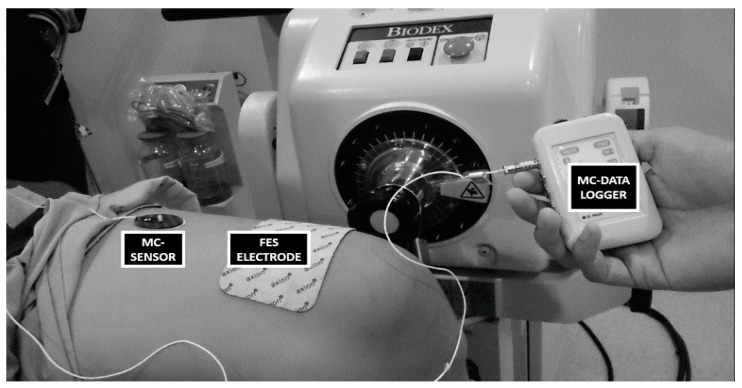
SCI individual seated on the dynamometer during FES-evoked contractions. The placement of neuromuscular stimulating electrodes and MC sensor over the subject’s rectus femoris muscle is shown.

The experimental setup is illustrated in Figure 1. FES characteristics were preset to 200 μs pulse width at 35 Hz pulse frequency, biphasic rectangular waveform and in the range of 70 mA to 110 mA current amplitude, to produce ‘vigorous’ leg muscle contractions. Two transcutaneous gel-backed neuromuscular stimulation electrodes (5 cm × 9 cm; RehaTrode, Hasomed GmbH, Magdeburg, Germany) were affixed over the subjects’ rectus femoris muscle belly and were connected to the stimulator (Figure 2). The MC-system assessed muscle tension using a commercial MMG sensor (TMG-BMC Ltd., Ljubljana, Slovenia). The neuromuscular stimulating electrodes were placed at the end of muscle origin and insertion, detected via manual palpitation of the muscle and confirmed with evoked stimulation to ensure maximum coverage of muscle stimulation, while the MC-sensors were affixed over the subject’s rectus femoris muscle belly. The basic structure of the MC Sensor consists of the sensor tip, silicon piezoresistive strain gauge and supporting part. The sensor is constructed in such a way that its pressure on the subject’s skin causes the sensor tip to compress the skin surface and the intermediate layer, ultimately placing pressure on the measured skeletal muscle. The sensor tip and strain gauge assembly is sensitive to any mechanical force change initiated by muscle contraction, transmitted through the skin above [16]. The force obtained from the MC sensor would be equal to:

Fs = 2F × cos α

**Figure 3:**
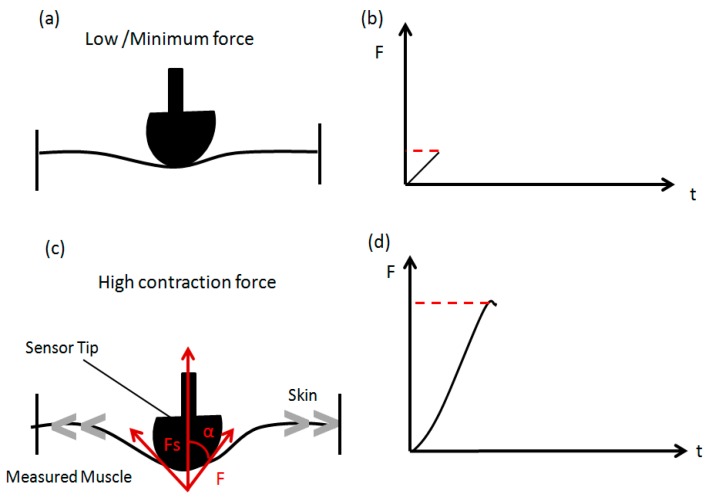
Working principle of the MC sensor in detecting variation in muscle force during contraction. (**a**,**b**) Measured force signal during low contraction (**c**,**d**) Measured force signal during high contraction.

The measured muscle tension produces the force F in the direction along the muscle surface. This force causes the intermediate layer and skin to press on the sensor tip (Figure 3). The vector sum of all forces produces the force Fs in the direction along the sensor tip and α is the angle between the directions if F and Fs [16]. Collected measurements data from the MC date logger can be analyzed and exported using Sensmotion software (TMG-BMC Ltd., Ljubljana, Slovenia).

Changes of muscle tension during FES-evoked contractions were measured, and the difference in muscle contraction intensities resulted in different amounts of muscle tension. The MC sensor was placed on the skin surface above the rectus femoris muscle belly using double-sided adhesive patches that enabled fixation of sensors to the subject’s skin (Figure 2). The sensor tip, pressed onto the skin surface, resulted in skin deformation over the muscle, and the muscle force, in turn, was exerted back to the sensor. Thus, the force measured at the sensor tip (measured in Vdc) was considered proportional to muscle tension evoked during FES [16]. The subject was seated on a Biodex Dynamometer System 4 Pro (Biodex Medical Systems, Shirley, New York, NY, USA) with their back supported, and knee joint angle fixed at 30° using the dynamometer attachment (knee adapter). Measurements were performed on the rectus femoris muscle (Figure 2) for both FES-evoked muscle performance and muscle fatigue components of the study.

### 2.3. Experimental Protocol

Each subject participated in two parts of the experimental design. The first part sought to investigate the relationship between of muscle-evoked isometric dynamometer torque (dyna-torque) and MC-sensor signal intensity. This was to determine whether the MC was responsive to different “intensities” of muscle performance during FES-recruitment of the rectus femoris in the SCI population. The second part investigated the sensitivity of the MC sensor as a potential proxy of muscle performance and fatigue. In this trial, the fatigue-related decrease of torque over a series of repetitive isometric muscle contractions is correlated with simultaneous MC sensor data.

#### 2.3.1. Part 1: Muscle Performance Trial

Participants attended five sessions of isometric muscle contractions, whereby different levels of neurostimulation current amplitude (in mA) were delivered for each FES-evoked contraction in a randomized order, within each set of the session. Five 2 s muscle contractions were evoked over 3–5 min with 30 s recovery between each repetition. The FES stimulation current amplitude was varied within in the range of 70 mA to 110 mA while the dyna-torque and MC signal voltage were collected. Randomization of stimulation current amplitude amongst sets was performed to prevent selection bias and to minimize fatigue effects. The testing protocol is illustrated in Table 2.

**Figure 4:**
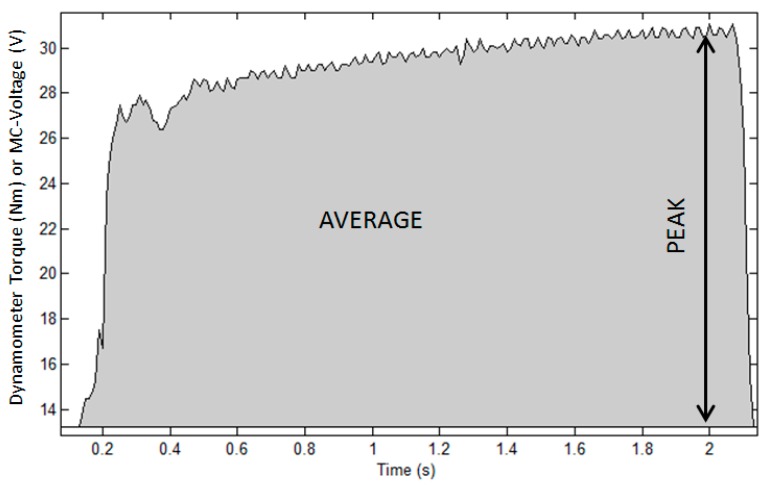
Schematic representation of a single FES-muscle contraction for derivation of dynamometer peak torque and average torque.

From the Biodex dynamometer, two variables were extracted to characterize muscle performance. Dynamometer peak torque was the highest knee-extension torque observed in each muscle contraction (Figure 4). Dynamometer average torque was expressed as the ‘area under the torque-time curve’ for each muscle contraction (Figure 4).

From the MC-Voltage for each FES-induced muscle contraction, two variables were derived—Signal Peak (SP) and Signal Average (SA). SP was the highest amplitude of the MC signal (V), and SA was the area under the voltage-time curve for each muscle contraction. These were investigated as possible proxies for dynamometer peak torque and average torque, respectively. To derive a prediction equation between SP and SA from MC-voltage, and the respective dynamometer peak and average torques (N·m), “bootstrapping” statistical analysis was performed. Initially, 40% of the dataset, stratified by current amplitude (Table 2), was randomly selected to derive an initial relationship for each individual fitting the linear regression:

**T**_(SP/SA)_ = β_0_ + β_1_∙MC_(SP/SA)_

where:

T = dynamometer torque (N·m)
β_0_ = y-intercept
β_1_ = slope of the line
MC = sensor output (V)
SP = Signal Peak
SA = Signal Average

Thence, for each individual’s regression equation, muscle torques (predicted MC torque) was estimated from MC-sensor voltages from the whole data set, for each subject.

#### 2.3.2. Part 2: Muscle Fatigue Trial

Following a minimum 15-min recovery after the first trial, subjects performed up to thirty FES-induced consecutive 4 s isometric contractions at 30° knee joint angle on the Biodex dynamometer with 10 s recovery between each repetition, while dynamometer torque (dyna-torque) and MC signal voltage were measured. For the muscle fatigue protocol, FES current amplitude was held constant at 100 mA, which was 91% of the highest tolerable neurostimulation current for the participants. Other FES characteristics (e.g., pulse duration, frequency, waveform shape) were the same as the first trial.

### 2.4. Data Analysis

The MC-voltage signal was sampled at 5 kHz and low-pass filtered with a cutoff frequency 10 Hz using MatLab (Mathworks Inc., Natick, MA, USA) [16]. To determine the relationship between the torque measured on the dynamometer and the MC signal voltage during both experiments, the signal peak (SP) and signal average (SA) were compared to the peak torque and average torque for each contraction. Before descriptive statistical analyses and regression modeling were performed, the dyna-torque and MC voltage data were normalized to each individual’s maximum muscle stimulation contraction for the simultaneous analysis of dyna-torque and MC voltage signals. Then, the mean absolute percentage errors (MAPE) between dynamometer measured torque and MC-predicted torque were calculated:

MAPE (%) = mean difference dyna-torque and predicted MC torque × 100/mean dyna-torque.

Consequently, the degree of association between isometric knee extension torque and predicted MC torque SP/SA was evaluated with the Coefficient of Determination (R^2^). Finally, to investigate the level of agreement between the SP/SA dyna-torque and SP/SA-predicted MC torque, Bland-Altman plots were constructed with 95% confidence limits of agreement and both bias (mean difference) and Lin’s concordance correlation coefficient (*ρ*_C_) were calculated. All data were analyzed and plotted using Microsoft Excel (Microsoft Corporation, Redmond, WA, USA) and SigmaPlot 13.0 (SYSTAT Software Inc., San Jose, CA, USA).

## 3. Results

### 3.1. Muscle Performance

**Figure 5:**
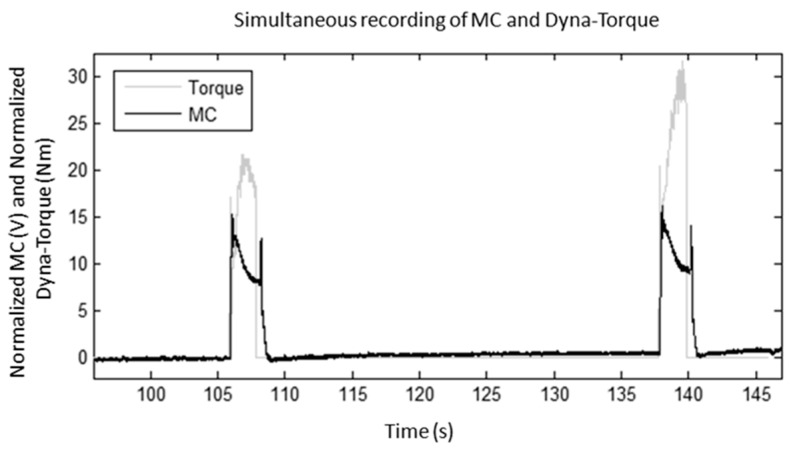
Normalized MC-voltage and isometric knee torque during FES-evoked contraction from one SCI individual.

Simultaneous measurement of exemplar normalized dyna-torque and normalized MC voltage signals revealed that the amplitude of MC rose concurrently with the increase of FES-evoked muscle torque (Figure 5). When the muscle contraction was elicited by the neuromuscular stimulator, the MC recorded a change in tension in the rectus femoris muscle around the knee joint. One participant’s data was not included in the statistical analyses, because of ‘outlier’ findings, however this data has been deliberated upon in the Discussion.

**Figure 6:**
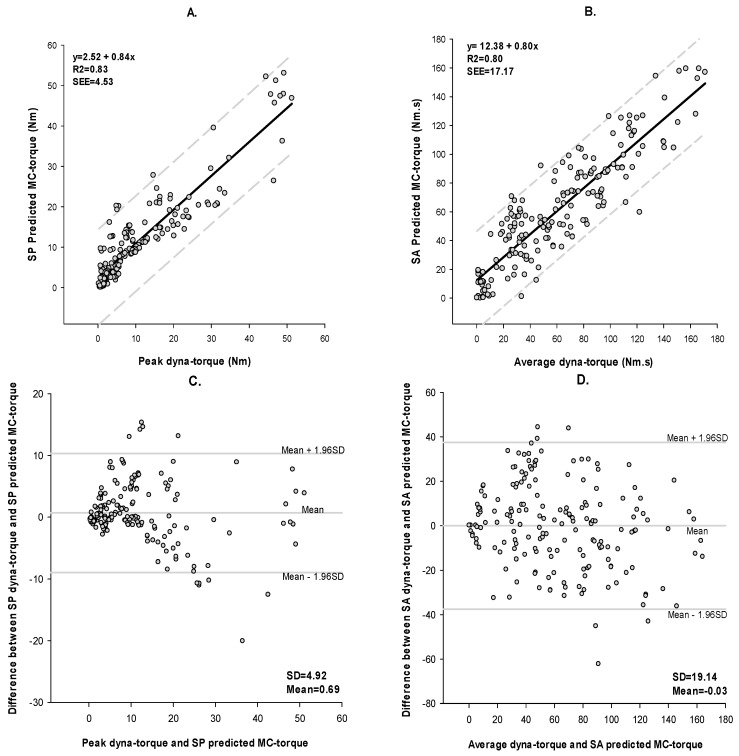
Linear regression and Bland-Altman plots between dyna-torque and the predicted MC torque. (**A**) Linear regression of peak dyna-torque and the MC-torque SP with 95% confidence bands; (**B**) Linear regression scatter plots of average dyna-torque and the MC-torque SA with 95% confidence bands; (**C**) Bland-Altman plots of peak dyna-torque and the Predicted MC-torque SP; (**D**) Bland-Altman plots of average dyna-torque and the Predicted MC-torque SA. Derivation of these variables has been described in the text.

In Figure 6, a comparison between the measured peak/average dyna-torques and the predicted-SP/SA MC torques were shown to have a strong linear relationship with a high degree of association (peak dyna-torque with SP MC torque r = 0.91, *p* < 0.0001; average dyna-torque with SA MC torque r = 0.89, *p* < 0.0001). Figure 6 also revealed that the MC sensor could measure muscle contraction values at low stimulation currents even when no dyna-torque could be recorded. As Bland-Altman plots portrayed in Figure 6C,D and Table 3, the measured peak and average dyna-torques and predicted-SP/SA MC torques had small bias and standard deviation (SD), which were SP: bias = 0.69; SD = 4.92, SA: bias = −0.03; SD = 19.14, and moderate-high concordance (*ρ*_C)_. MAPE was in the moderate range.

### 3.2. Fatigue

**Figure 7:**
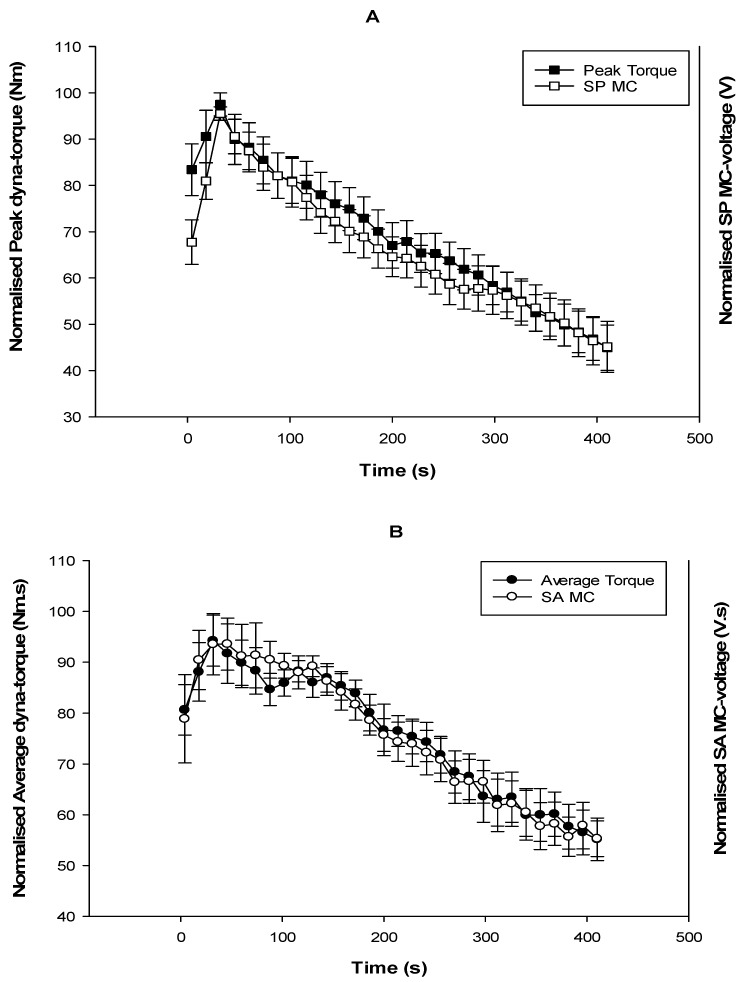
Rectus femoris fatigues in SCI participants represented by their mean normalized dyna-torque and mean normalized MC voltages during FES-evoked contractions (**A**) Peak dyna-torque and SP MC voltage throughout 30 contractions over 400 s; (**B**) Average dyna-torque and the SA MC-voltage throughout 30 contractions over 400 s. Data are mean ± standard error.

The second protocol involved 30 repetitive 4 s FES-evoked contractions at 100 mA current amplitude. Figure 7 portrays the mean values of normalized peak/average dyna-torques and normalized SP/SA MC-voltages in the nine SCI participants. During the foregoing FES-evoked muscle contractions, the peak torques and average torques with corresponding and MC-voltage SP/SA decreased. After the first three FES-evoked contractions of their rectus femoris muscles, rapid fatigue was observed in the SCI individuals.

**Figure 8:**
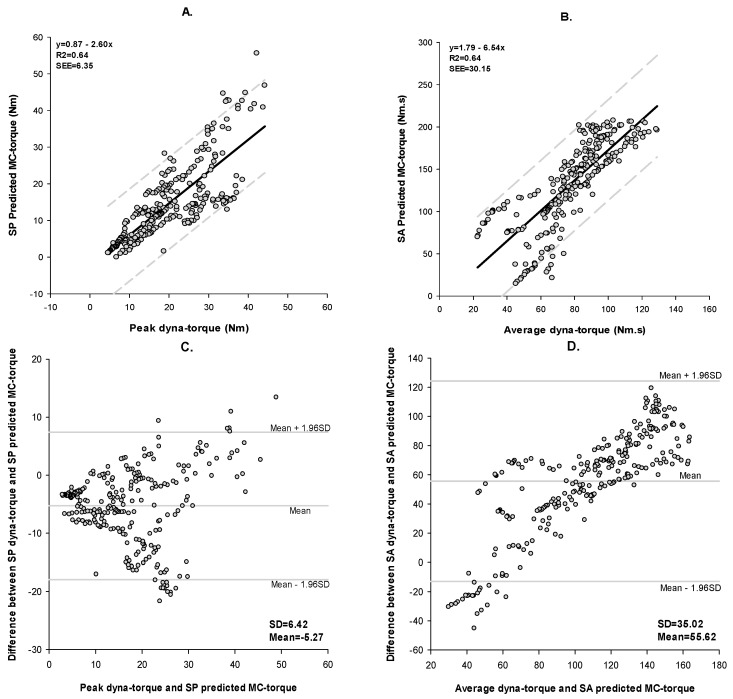
Linear regression and Bland–Altman plots between dyna-torque and the predicted MC torque during the muscle fatigue experiment. (**A**) Linear regression of Peak dyna-torque and the SP predicted MC torque with a 95% confidence band; (**B**) Linear regression of Average dyna-torque and the SA predicted MC torque with a 95% confidence band; (**C**) Bland–Altman plots of Peak dyna-torque and the SP predicted MC torque; (**D**) Bland–Altman plots of Average dyna-torque and the SA-predicted MC torque.

The high correlation between dynamometer peak torques during fatiguing muscle contractions and SP MC-predicted torques (Figure 8A; r = 0.80; *p* < 0.0001), and between dynamometer average torques over time and SA MC torques (Figure 8B; r = 0.77; *p* < 0.0001), suggested the MC sensor well-represented FES-evoked muscle fatigue, acting as a close proxy for dynamometer measurements. From the Bland-Altman plots, Figure 8C,D and Table 3, the measured peak/average dyna-torques and predicted SP/SA MC torques with bias and standard deviation, were SP: 5.27 (6.47); SA: 55.62 (35.02); and fair-moderate concordance (*ρ*_C_). MAPE was in the fair-moderate range. Some ‘trend-biases’ were visually noted in BA analysis of peak/average dyna-torques and the respective SP/SA MC estimated torques during the muscle fatigue trial.

## 4. Discussion

Prolonged FES-evoked contractions in individuals with SCI during their post-SCI rehabilitation may lead to rapid fatigue, whereby their muscles are not able to maintain a constant work production due to decrease in muscle force [9,21]. The condition of rapid-onset muscle fatigue may even lead to muscle damage [22]. Even though there are other muscle fatigue monitoring techniques including dynamometer, EMG, NIRS and other force sensors currently available, the MC sensor may be a practical choice for clinicians due to its reliability and ease of use compared to other techniques [23,24]. Such a use of an MC sensor has been previously proposed by Dordevic and colleagues [16], who demonstrated a strong correlation between MC voltage and force in 21 able-bodied subjects (R^2^ = 85%). However, the MC sensor as fatigue proxy, especially during FES-evoked contractions in SCI patients, has not been previously investigated. Thus, the findings of this current study are the first application of the MC sensor for estimating muscle peak forces and muscle fatigue rates in a neurological population.

### 4.1. Method of Validation

This study sought to determine the sensitivity and accuracy of MC as muscle contraction sensor and determine whether it might be a good fatigue proxy for FES-evoked muscle contractions. It was observed that even though the start and end time, as well as the duration of MC and torque signal, were the same, the peak values of MC and torque were not simultaneous. The nature of pattern of each signal decline were observed to behave differently, even though they rise at the same time (Figure 5). We reported a strong dyna-torque vs predicted MC torque relationship, with r = 0.91 (R^2^ = 83%) in muscle performance over a range of FES current amplitudes and r = 0.80 (R^2^ = 64%) under conditions of progressive muscle fatigue. This linear relationship demonstrated that the MC sensor could be used as a suitable proxy for muscle tension for individuals with SCI undertaking FES rehabilitation training, and established a satisfactory representation of muscle performance during progressive fatigue. While the signal was highly correlated with the measured dyna-torque, there was some variability that might have been due to the skin and underlying adipose tissue of different individuals. Additionally, in the fatigue experiment, some subjects’ dynamometer measurements could not be detected, yet the MC sensor still recorded skin-muscle deformation of muscle contraction. A possible cause may have been the thickness of skin tissue or that some individuals with SCI might have fatigued earlier than others, thus no contraction torque could be detected by the Biodex dynamometer. Figure 7 shows the increment of the dynamometer torque and MC voltage in the first 3 initial contractions (meaning an increase in strength), and the rapid decrease of torque values observed in the results (fatigue). This is because it takes a short period of time for the muscles of a paraplegic to reach their peak force-generating capacity under FES. Similar results were also shown in previous studies [8,21,25].

### 4.2. Method of Agreement: Bland-Altman

Traditional regression analyses revealed a strong linear correlation between dyna-torque and the predicted MC torque, however this does not necessarily translate into a strong case for agreement between the two techniques. Thus, Bland and Altman [26] analyses were performed to quantify the degree of agreement between the two methods of measuring muscle contraction (i.e., using dynamometer and the MC sensor).

There was a strong agreement between of dyna-torque and predicted MC torque during muscle performance over a range of FES current amplitudes, with *ρ*_C_ = 0.91 for SP and *ρ*_C_ = 0.89 for SA, respectively. Figure 6C,D illustrated the difference in muscle contraction torque measured by a dynamometer versus predicted by the MC sensor. Across the range of muscle forces measured in these subjects, the mean difference (SP/SA dyna-torque—SP/SA-predicted MC torque) were 0.69 for SP and −0.03 for SA. Most of the measured muscle contractions were clustered close to the mean difference line, and lay within the 95% limits of agreement, suggesting a strong trend for agreement between dyna-torque and predicted MC torque [27]. Indeed, the limits of agreement for both SP and SA were narrow enough for one to be confident that the small body-worn MC sensor could be used in the place of a large laboratory-based dynamometer for measuring muscle contractions within a clinical environment. Under conditions of progressive muscle fatigue, the agreement analysis between the peak/average dyna-torque and SP/SA predicted MC torque revealed *ρ*_C_ = 0.70 for SP, but was quite low for SA at *ρ*_C_ = 0.30. Even though the measured contractions were clustered within ± 1.96 SD of mean difference (Figure 8D), the 95% limits of agreement were much wider for SA (between −13.01 and 124.26 N·m), crossing cipher, which suggested that that high intra-individual and between-subject variability rendered the MC sensor less acceptable for estimating peak and average torques during muscle fatigue, in our sample of SCI individuals. In addition, visual inspection of the BA analyses for the fatigue trials (Figure 8C,D) revealed “fan-spread bias” and “linear-bias” in the data. This suggested that the difference between measured dyna-torque and predicted MC torque during the muscle fatigue trial (comprising thirty FES-induced consecutive 4 s isometric contractions) varied with the magnitude of torque output.

### 4.3. Mean Absolute Percentage Error (MAPE)

The MAPE measured the prediction “accuracy” of signal peak (SP) and signal average (SA) from MC torque. With MAPE ranging from 49% to 77% during muscle performance and muscle fatigue experimental trials, there were moderate-good levels of prediction for dyna-torque in these individuals. These findings suggested that the small MC sensor might be more sensitive under low FES current amplitudes and low torque; a conventional laboratory dynamometer in recording evoked muscle contractions in persons with SCI. Our findings also revealed that the MC-sensor might also be used as a muscle fatigue monitor (but noting the ‘bias’ caveat previously stated), adding its utility to the other commonly used sensors of fatigue such as sEMG and vibromyography [10,11]. Since MC sensor measurements are based on skin-muscle tension, its detection threshold was low enough to quantify ‘weak’ contractions, (portrayed in Figure 6), even under conditions of low torque muscle activity when the dynamometer could not.

**Figure 9:**
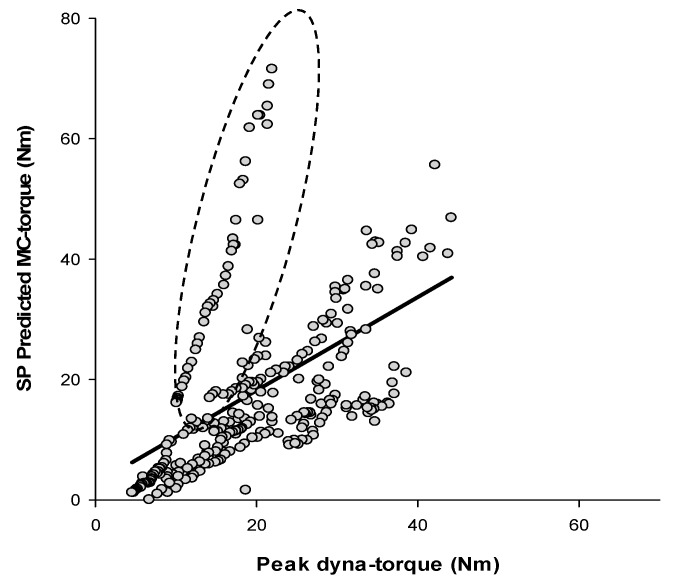
Regression analysis between Peak dyna-torque and SP predicted MC during the muscle fatigue experiment; includes 1 subject (data in dashed circle).

In this current study, one of the 9 SCI participants had a history of neuromuscular ‘spasticity’. This individual demonstrated ‘different’ predicted muscle torque based on the MC sensor from the fatigue trial (Part 2) than from the performance trial (Part 1). This ‘outlier’ finding suggested an additional caveat, whereby the MC sensor may lead to inaccurate estimates of “true” muscle forces in individuals with particular muscle conditions such as spasticity, rigidity or contracture. Figure 9 illustrates that this particular subject’s predicted MC data during the fatigue trial portrayed a slope much steeper than the other subjects. The reason might have been muscle stiffness or torpor, which may have been due to lack of regular stretching and/or FES-training before the experiment was conducted. Yet, even this ‘unusual’ MC relationship managed to distinguish and estimate the difference in low torque production.

One advantage of the MC sensor over other muscle sensors is that MC data is relatively easy to analyze because its measurement is based on muscle tension changes, the signal pattern correlates directly with the instantaneous torque profile (portrayed in Figure 5) compared to other MMG signals [28]. Additionally, the MC signal is not hampered by any electrical properties in contact between the skin and the electrode [29,30]. Tarata and co-workers [13] reported that the mechanical components of the muscle (such as torque and contraction speed) are more closely related to the muscle function than to its electrical characteristics. Thus, the measurement of muscle activity does not require such a complex laboratory setup as the Biodex dynamometer, and the SCI users’ muscle performance could be monitored over a wider range of daily rehabilitation and physical therapy tasks with the MC sensor, especially FES-evoked standing where the muscle contraction is isometric in nature, and other dynamic movement of the muscles following further research on MC sensor applications. Contrariwise, the MC is a relatively new sensor, and at the time of this current study, the MC-sensor’s data could only be saved in a data logger and not be transferred wirelessly in real-time during the study. Future technical developments must achieve wireless, real-time portrayal of MC peak and average estimates of muscle performance, for clinical efficacy. Different protocols to validate the MC-sensor as muscle performance and muscle fatigue monitor with a greater number of SCI participants and in different settings are also recommended for future investigation, including the effects of environmental factors such as temperature and humidity.

## 5. Conclusions

In conclusion, the strong linear correlation between MC sensor torque measurements and dynamometer isometric knee torque suggested that the MC sensor was able to detect different muscle contraction levels and a fatiguing contraction in FES-evoked activity among individuals with SCI.

## Figures and Tables

**Table 1:** Physical Characteristics of SCI study participants.

Subject	Age (year)	Gender	Height (m)	Body Weight (kg)	Spinal Lesion Level	Injury Classification (AIS *)	Years Since Injury (year)
1	28	M	1.71	62.4	C7	B	14
2	35	M	1.61	81.0	T10–T11	A	18
3	59	M	1.79	71.6	C6–C7	B	2.5
4	47	F	1.62	82.0	T4	B	24
5	58	M	1.73	80.0	C5–C7	B	4
6	33	M	1.71	44.0	C5–C6	A	13
7	59	M	1.72	60.5	T10–T11	A	13
8	34	M	1.70	75.9	C6	A	17
9	21	F	1.60	48.0	T10	B	1

* “AIS” denotes the American Spinal Injuries Association Impairment Scale [20].

**Table 2:** Schema illustrating the muscle stimulation current protocol (mA).

	Session	1	2	3	4	5
Set						
1		70	80	90	100	110
2		80	90	100	110	70
3		90	100	110	70	80
4		100	110	70	80	90
5		110	70	80	90	100

**Table 3:** Muscle performance and muscle fatigue analysis for signal peak and signal average relationships of dyna-torque and predicted MC torque.

Experiment Outcome	N	*p*	R^2^	SEE	Bland-Altman Bias	*ρ_C_*	MAPE
Performance							
SP	225	<0.0001	83%	4.53	0.69	0.91	70%
SA	225	<0.0001	80%	17.17	−0.03	0.89	70%
Fatigue							
SP	304	<0.0001	64%	6.35	−5.27	0.70	49%
SA	304	<0.0001	64%	30.15	55.62	0.30	77%

N—Total; *p*—*p* value; R^2^—Correlation of determination; SEE—Standard Error Estimate; *ρ*_C_—Lin’s Concordance; MAPE—Mean Absolute Percentage error.
